# Clinical study on the effect of low-intensity pulsed ultrasound on healing of proximal sesamoid bone fractures in Yili horses

**DOI:** 10.1038/s41598-025-17424-0

**Published:** 2025-08-28

**Authors:** Zhiyuan Zhang, Jianlong Li, Zhanhai Mai, Yang Yang, Han Fu, Xiongjian Cao, Tianqing Li, Qingyong Guo, Yuhui Ma

**Affiliations:** 1 College of Animal Medicine, Xinjiang Agricultural University, Urumqi, 830052 Xinjiang China; 2 XingJiang Zhaosu County Xiyu Horse Industry Co., Ltd, Yili, 835600 Xinjiang China

**Keywords:** Yili horses, Proximal sesamoid bone, Low-intensity pulsed ultrasound, Radiographic diagnosis, Imaging, Bone, Diagnostic markers

## Abstract

The incidence of sports injuries in horses is increasing, thus accurate assessment, diagnosis, and treatment are critical. Among common sports-related injuries, proximal sesamoid bone fractures (PSBFs) are one of the most frequent types. To investigate the effects of low-intensity pulsed ultrasound (LIPUS) on imaging and hematological parameters of PSBFs, providing a clinical reference for the diagnosis and treatment of PSBFs in racehorses. After clinical diagnosis and radiographic examination confirmed the disease and its location, the affected horses were randomly divided into two groups: the ultrasound group received daily LIPUS treatment, while the control group received no intervention. After a four-week treatment, lameness grading, imaging, and hematological parameters were reassessed to evaluate PSBF healing and changes in blood indicators. Both imaging and hematological examinations play important clinical roles in the early diagnosis and later-stage evaluation of diseases. In this study, following LIPUS treatment, the affected horses showed significantly increased ALP activity in the serum, and higher levels of serum calcium (Ca), phosphorus (P), and the calcium-phosphorus product (Ca × P) (*P* < 0.05). Additionally, analysis of inflammatory cytokines revealed that the expression of pro-inflammatory markers, TNF-α, IL-1β, and IL-6, were markedly elevated during the early stages of fracture but gradually returned to normal as treatment progressed. These findings suggest that LIPUS can accelerate healing by alleviating inflammation at the fracture site. This study found that LIPUS treatment of PSBFs significantly improved microcirculation at the fracture site and promoted efficient healing through enhanced callus formation and accelerated bone repair. This favorable outcome of LIPUS therapy provides a valuable clinical reference for future diagnosis and treatment of PSBFs.

**Supplementary Information:**

The online version contains supplementary material available at 10.1038/s41598-025-17424-0.

## Introduction

Yili horses are a versatile breed developed in China for both riding and draft purposes. They are primarily found in the Yili Kazakh Autonomous Prefecture of Xinjiang. The breeding of Yili horses began in the twentieth century, with Kazakh horses serving as the maternal foundation. Excellent light horse breeds such as Orlov Trotter, Don, Budyonny, and Akhal-Teke were introduced through crossbreeding. The breed was developed under long-term grazing conditions, resulting in a new type characterized by large stature, excellent athletic performance, strong meat and milk production capabilities, tolerance to coarse feed, and high adaptability to harsh environments^1–3^. Clinical practice has shown that among limb injuries to Yili horses, the incidence of proximal sesamoid bone fractures (PSBF) is relatively high. The proximal sesamoid bones (PSBs) are a pair of pyramid-shaped bones located on the palmar/plantar aspect of each metacarpophalangeal/metatarsophalangeal (fetlock) joint. Besides their dorsal articular surfaces, PSBs are embedded in extensive ligament attachments, which together form the suspensory apparatus^4^. The PSBs articulate with the distal palmar/plantar end of the third metacarpal/metatarsal bone (MC3/MT3) and serve both as fulcrums for the suspensory apparatus and as smooth gliding surfaces for the flexor tendons. Catastrophic fractures of the PSBs can cause failure of suspensory apparatus and are among the most common fatal musculoskeletal injuries in Thoroughbred racehorses in the United States and Hong Kong^5–9^. PSBF is a leading cause of euthanasia in Thoroughbred racehorses in the U.S.^10,11^. Low-intensity pulsed ultrasound (LIPUS) is an emerging therapeutic modality that has garnered attention due to its non-invasive nature and multiple biological effects^12^. LIPUS therapy has been widely recognized for its ability to promote endochondral ossification^13^. It increases blood flow near the injury site and shortens the healing time of sesamoid bone fractures, tibial fractures, and distal radial fractures^14^. The therapeutic ultrasound used in LIPUS is safe and does not require surgical intervention^15^. When applied during the initial phase of fracture, LIPUS can achieve higher treatment efficiency^16^.

LIPUS emits low-intensity (< 3 W/cm^2^), pulsed ultrasound waves (0.7–3.0 MHz), delivering non-traumatic mechanical forces. These mechanical and cavitation effects can promote endothelial cell regeneration, suppress inflammatory responses, reduce oxidative stress, and protect myocardial cells, thereby demonstrating significant clinical value and scientific potential in the treatment of cardiovascular diseases (CVDs)^17,18^. In fields such as orthopedics and urology, LIPUS has shown remarkable efficacy in accelerating bone fracture healing and promoting soft tissue repair^19,20^. With the increasing popularity of equestrian competitions in China, events across Xinjiang have become more frequent, leading to a rise in exercise-related injuries among horses. In this context, accurate injury assessment and timely diagnosis by equine veterinarians play a crucial role. This study applied LIPUS to treat PSBFs in Yili horses and conducted lameness assessments, imaging analyses, and hematological tests to evaluate disease progression and physiological responses, thereby offering valuable reference data for the diagnosis and treatment of PSBFs in horses worldwide.

## Results

### Clinical examination before treatment

**Fig. 1:**
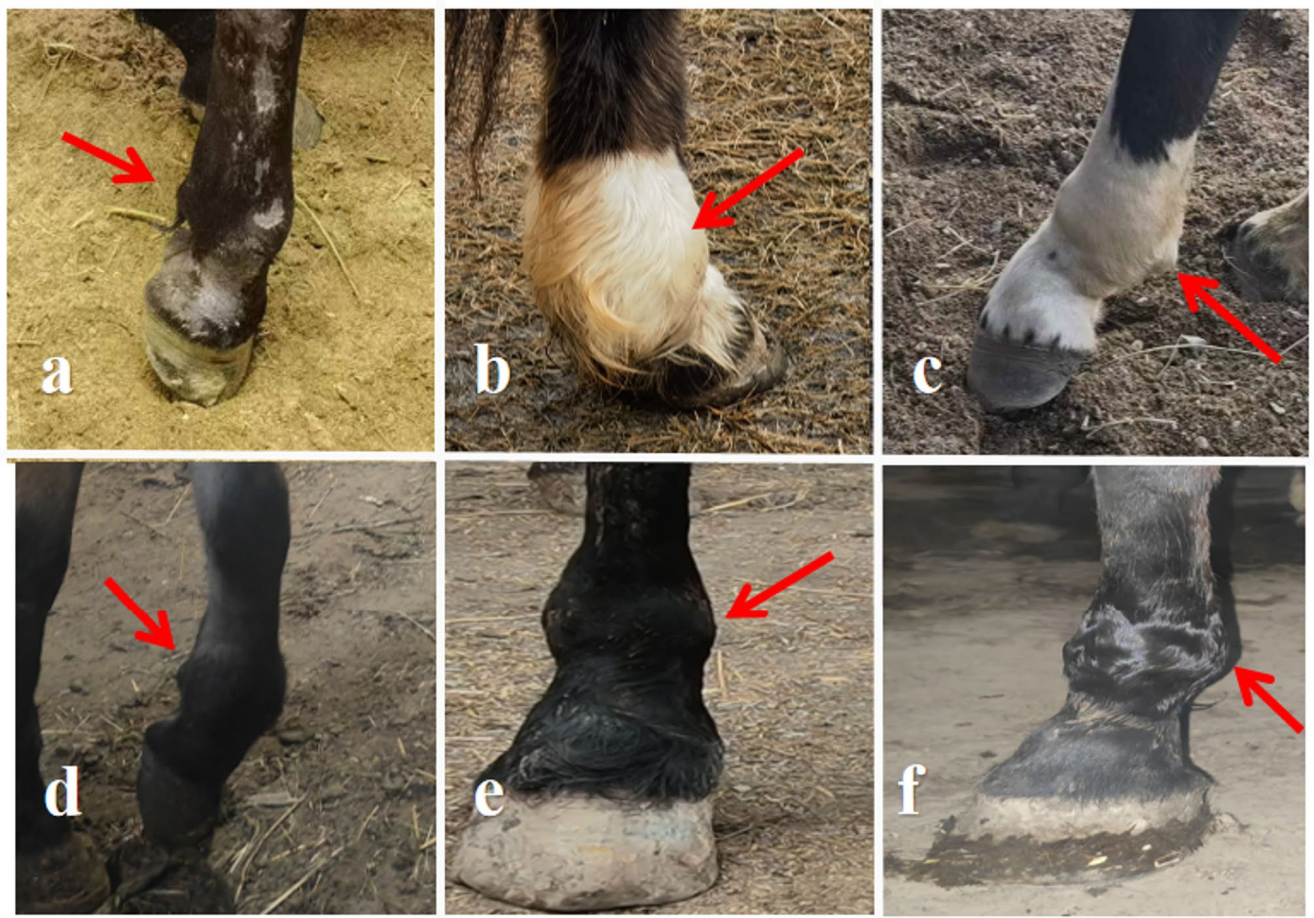
Clinical findings in the affected horse. (**a**-**c**) Incomplete swelling of the lateral tubercle of the lateral proximal sesamoid fracture. (**d**, **e**) Incomplete swelling of the medial tubercle of the medial proximal sesamoid fracture. (**f**) Complete swelling of the ball segment of the medial and lateral proximal sesamoid fracture.

Prior to LIPUS treatment, horses in both the ultrasound group and control group exhibited signs of mental restlessness, frequent stamping while standing, and evident lameness during lead walking. Swelling of the affected fetlock was pronounced, and palpation elicited a clear pain response (Fig. 1). Table 1 presents the baseline information of horses in the ultrasound, control, and normal groups before treatment.

**Table 1:** Basic information about the horse before treatment.

Basic information	Ultrasound therapy group	Control group	Normal group
Age/(Year)	2.7 ± 0.8	2.7 ± 0.5	2.8 ± 0.7
Weight/(Kg)	407 ± 15	418 ± 22	411 ± 16
Body temperature/(°C)	38.3 ± 0.4	38.2 ± 0.2	37.7 ± 0.3
Heart rate/(beats/min)	39 ± 3	39 ± 3	35 ± 3
Fetlock circumference/(cm)	27.2 ± 1.5	27.1 ± 1	24.4 ± 0.4

### Lameness scoring before treatment

**Fig. 2:**
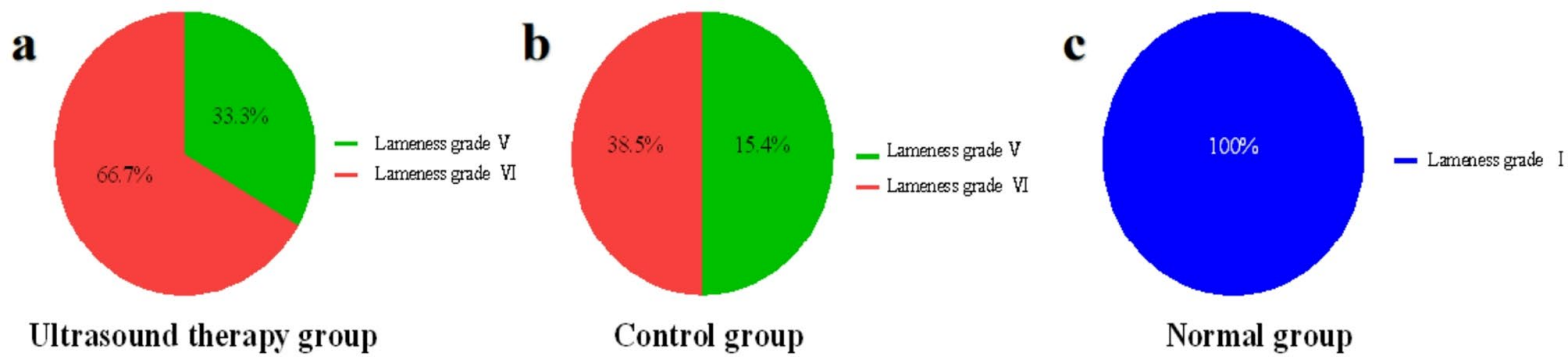
Classification of lameness in horses in each group.

Lameness evaluation and grading were conducted for horses in the ultrasound, control, and normal groups. As shown in Fig. 2, all affected horses were unilaterally lame. In the ultrasound group (Fig. 2a), lameness grading was: Grade I: 0 horses; Grade II: 0; Grade III: 0; Grade IV: 0; Grade V: 2; Grade VI: 4. In the control group (Fig. 2b): Grade I: 0; Grade II: 0; Grade III: 0; Grade IV: 0; Grade V: 3; Grade VI: 3. In the normal group (Fig. 2c): all 6 horses were Grade I, with no signs of lameness.

### Radiographic findings before treatment

Prior to LIPUS therapy, radiographic imaging was conducted on the affected horses in both the ultrasound and control groups to assess the type of PSBF in Yili horses. Imaging sites included the left forelimb (LF), right forelimb (RF), left hindlimb (LH), and right hindlimb (RH), with projection angles of lateromedial (LM), 45° dorsolateral–palmaromedial oblique (DL-PaMO), and 45° palmarolateral–dorsomedial oblique (PaL-DMO).

The proportions and representative radiographic images of different PSBF types are shown in Fig. 4: apical and avulsion fractures accounted for 50% (6/12), mid-body fractures for 17% (2/12), basal fractures for 25% (3/12), and mixed (apical + basal) fractures for 8% (1/12). Among these, apical and avulsion fractures were the most common.

**Fig. 3:**
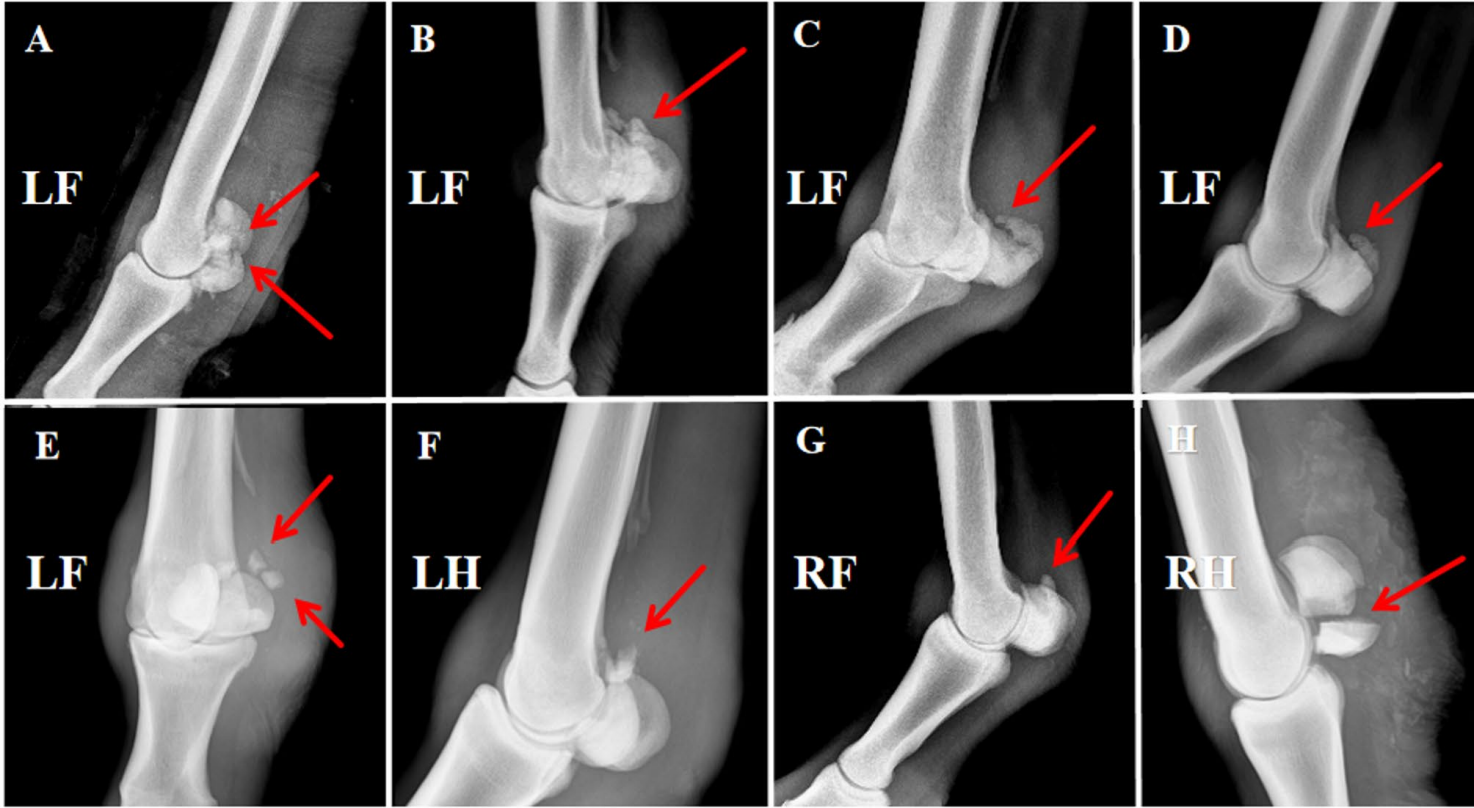
PSBF imaging findings. (**A**) mixed proximal sesamoid (apical + basal) fracture; (**B**) and (**C**–**E**) proximal apex sesamoid fractures; (**F**), (**H**) apex + base fracture of sesamoid bone; (**G**) proximal sesamoid base fracture; The red arrow in the figure shows the lesion.

In addition, radiographs showed that some horses developed varying degrees of sesamoiditis post-fracture (Fig. 3, panels A, B, F), with evidence of periosteal bone proliferation and even focal bone resorption.

### Clinical findings after treatment

**Fig. 4:**
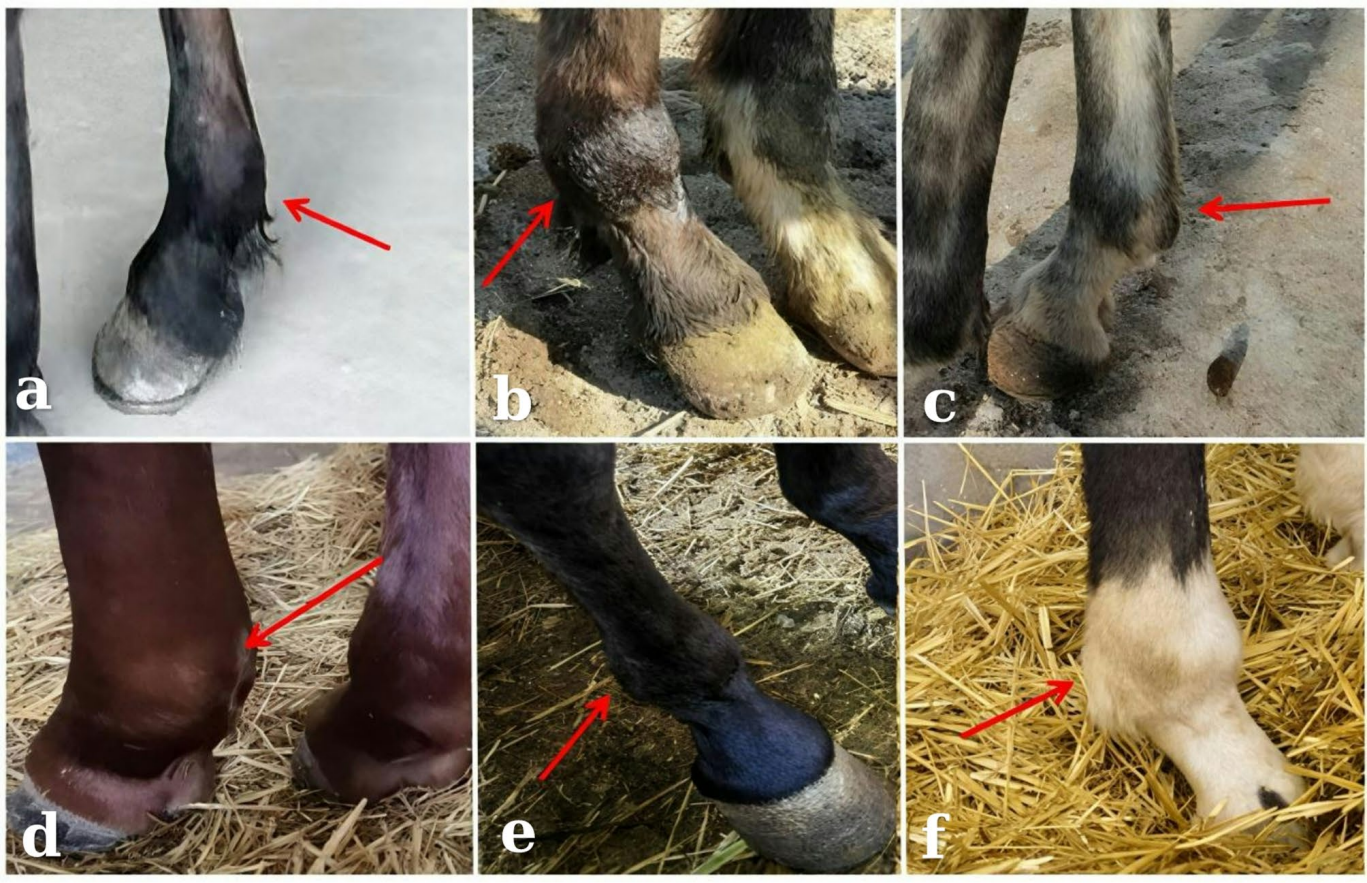
Clinical findings after treatment. (**a**–**c**) No more swelling at the proximal seed bone and ball joint after LIPUS treatment. (**d**–**f**) Complete swelling at the ball joint of the medial lateral proximal seed bone fracture.

After 4 weeks of LIPUS treatment, clinical assessments showed (Fig. 4): horses in the ultrasound group (Fig. 4a–c) exhibited good mental status, no visible lameness while standing, and marked improvement during lead walking. Swelling in the affected fetlock was reduced, and pain response to palpation was minimal.

In contrast, horses in the control group (Fig. 4d–f) remained mentally restless, showed frequent stamping while standing, and had persistent lameness during movement. Fetlock swelling and tenderness upon palpation were still apparent. Table 2 presents post-treatment data for the three groups.

**Table 2:** Basic information about horses after treatment.

Basic information	Ultrasound therapy group	Control group	Normal group
Age/(Year)	2.7 ± 0.8	2.7 ± 0.5	2.8 ± 0.7
Weight/(Kg)	416 ± 12	415 ± 18	418 ± 15
Body temperature /(℃)	37.8 ± 0.3	38.4 ± 1.2	37.7 ± 0.3
Heart rate /(beats/min)	38 ± 3	41 ± 2	34 ± 2
Fetlock circumference /(cm)	25 ± 0.6	26.8 ± 1.2	24.4 ± 0.4

### Lameness scoring after treatment

**Fig. 5:**
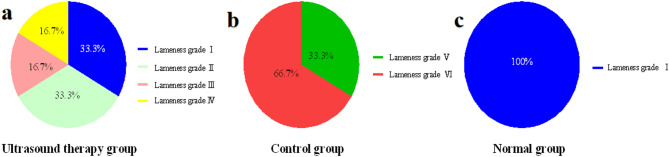
Classification of lameness.

Post-treatment lameness scores are shown in Fig. 5. In the ultrasound group (Fig. 5a): Grade I: 2 horses; Grade II: 2; Grade III: 1; Grade IV: 1; Grade V: 0; Grade VI: 0. In the control group (Fig. 5b): Grade I: 0; Grade II: 0; Grade III: 0; Grade IV: 0; Grade V: 2; Grade VI: 4. All horses in the normal group (Fig. 5c) remained Grade I.

### Radiographic findings after treatment

**Fig. 6:**
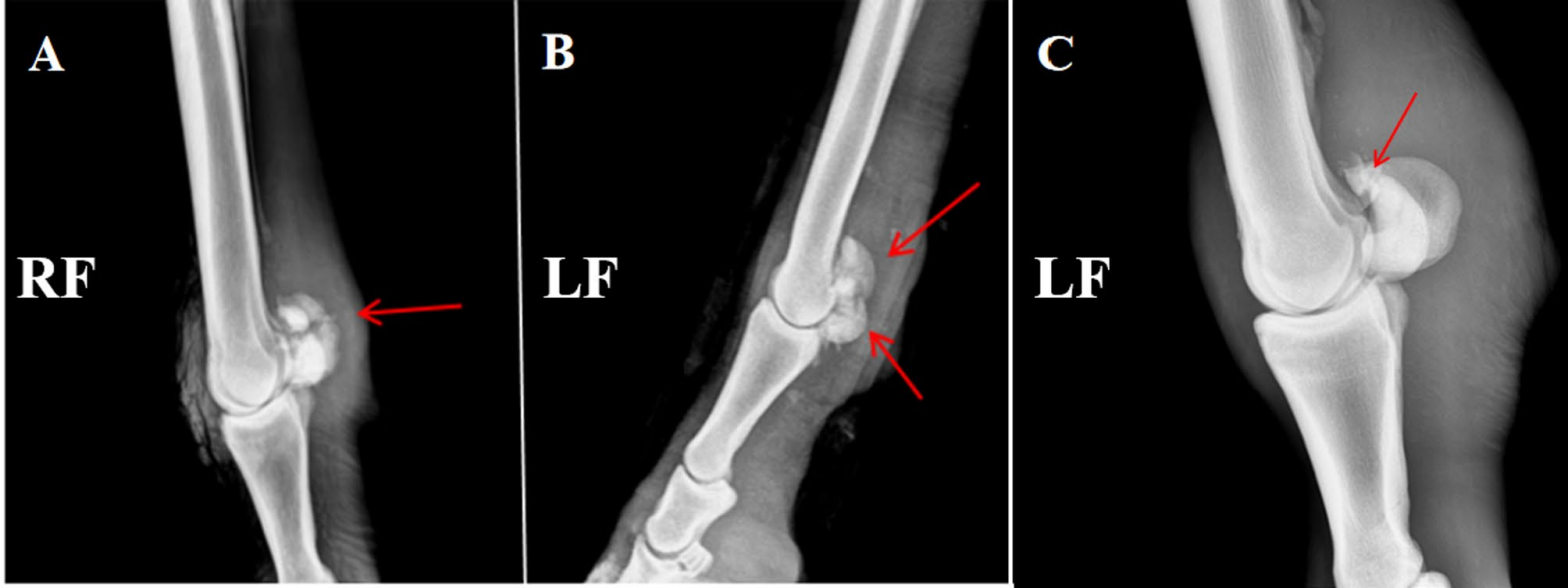
PSBF imaging images of each group after treatment. (**A**) Scabs formed by fractures of the proximal seed bone apices and avulsion fractures; (**B**) scabs formed by mixed (apical + basal) fractures; and (**C**) failure of the broken ends of the proximal seed bone apices to form a bony connection.

After 4 weeks of LIPUS therapy, follow-up X-ray imaging (Fig. 6) of the proximal sesamoid bone fractures (indicated by red arrows) revealed:

In the ultrasound group (Fig. 6A, B), the fracture site exhibited fibroblast and chondrocyte activity with formation of fibrous and cartilaginous callus, indicating early-stage bridging of the fracture ends.

In contrast, the control group (Fig. 6C) showed no formation of solid or continuous bony union at the fracture site.

### Hematological results

Hematological results are shown in Table 3. Compared to the normal group, the control group exhibited significantly elevated levels of NEU, WBC, and LYM (*P* < 0.05). RBC, HGB, MPV, and HCT were decreased; MPV and HCT showed significant differences (*P* < 0.05), while RBC and HGB showed highly significant differences (*P* < 0.01).

**Table 3:** Results of blood physiological indexes in each group.

Projects	Normal group	Control group	Ultrasound therapy group
WBC/(× 10^9^/L)	11.02 ± 0.46	12.20 ± 0.77	10.97 ± 0.71
NEU/(× 109/L)	6.70 ± 0.19	6.91 ± 0.22	6.70 ± 0.18
LYM/(× 10^9^/L)	5.71 ± 0.15	6.22 ± 0.52	5.79 ± 0.25
EOS/(× 10^9^/L)	0.57 ± 0.25	0.60 ± 0.24	0.60 ± 0.2
BASO/(× 10^9^/L)	0.06 ± 0.01	0.08 ± 0.02	0.07 ± 0.03
MONO/(× 10^9^/L)	0.57 ± 0.16	0.57 ± 0.18	0.56 ± 0.17
RBC/(× 1012/L)	6.02 ± 0.39	5.28 ± 0.33	5.94 ± 0.36
HGB/(g/L)	108.5 ± 5.24	99 ± 1.9	105 ± 3.9
HCT/(%)	37.3 ± 6.3	27.4 ± 5.1	35.4 ± 6.3
MPV/(fL)	6.24 ± 0.76	5.46 ± 0.02	6.07 ± 0.55

**Fig. 7:**
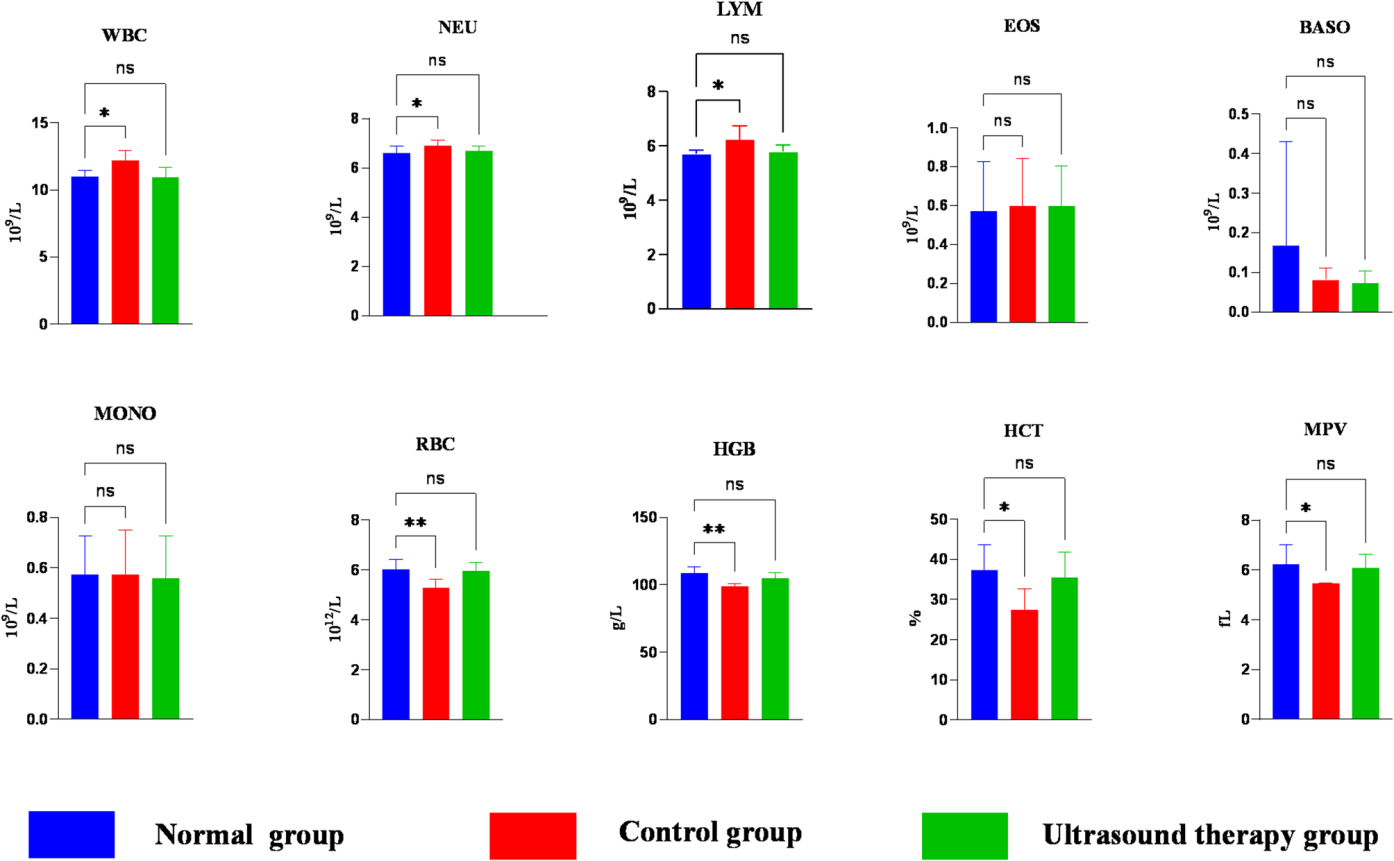
Differences in blood physiological indices between groups. WBC.white blood cells; NEU,neutrophils; LYM, lymphocytes; EOS, eosinophils; BASO, basophils; MONO, monocytes; RBCs, red blood cells; HGB, hemoglobin; HCT, hematocrit; MPV, mean corpuscular volume. One-way ANOVA was used to compare indices. In the bar graphs, asterisks indicate statistical significance: *P < 0.05; **P < 0.01; ***P < 0.001; ****P < 0.0001. In tables, lowercase superscripts denote *P* < 0.05 (significant difference); uppercase superscripts indicate *P* < 0.01 (highly significant difference); no superscript indicates *P* > 0.05 (no significant difference).

After 4 weeks of LIPUS treatment, no statistically significant differences were observed in hematological indices between the ultrasound and normal groups (*P* > 0.05) (Fig. 7).

### Biochemical results

Biochemical results are shown in Table 4. Compared to the normal group, the control group showed significantly elevated CK, LDH, and P levels (*P* < 0.05), while Ca × P, ALP, and Ca showed no significant differences (*P* > 0.05).

**Table 4:** Results of blood biochemical indices in each group.

Index	Normal group	Control group	Ultrasound therapy group
ALP/(U/L)	255.3 ± 42.3	281.8 ± 24.4	311.7 ± 45.5
CK/(U/L)	261.2 ± 18.7	296.58 ± 11.7	243.6 ± 29
LDH/(U/L)	441.6 ± 32.6	504.9 ± 45.2	423.6 ± 44.9
Ca/(mmol/L)	2.71 ± 0.08	2.94 ± 0.1	3.15 ± 0.4
P/(mmol/L)	0.92 ± 0.2	1.32 ± 0.31	1.58 ± 0.3
Ca × P/(mmol/L)	2.5 ± 0.6	3.66 ± 0.9	5.01 ± 1.4

**Fig. 8:**
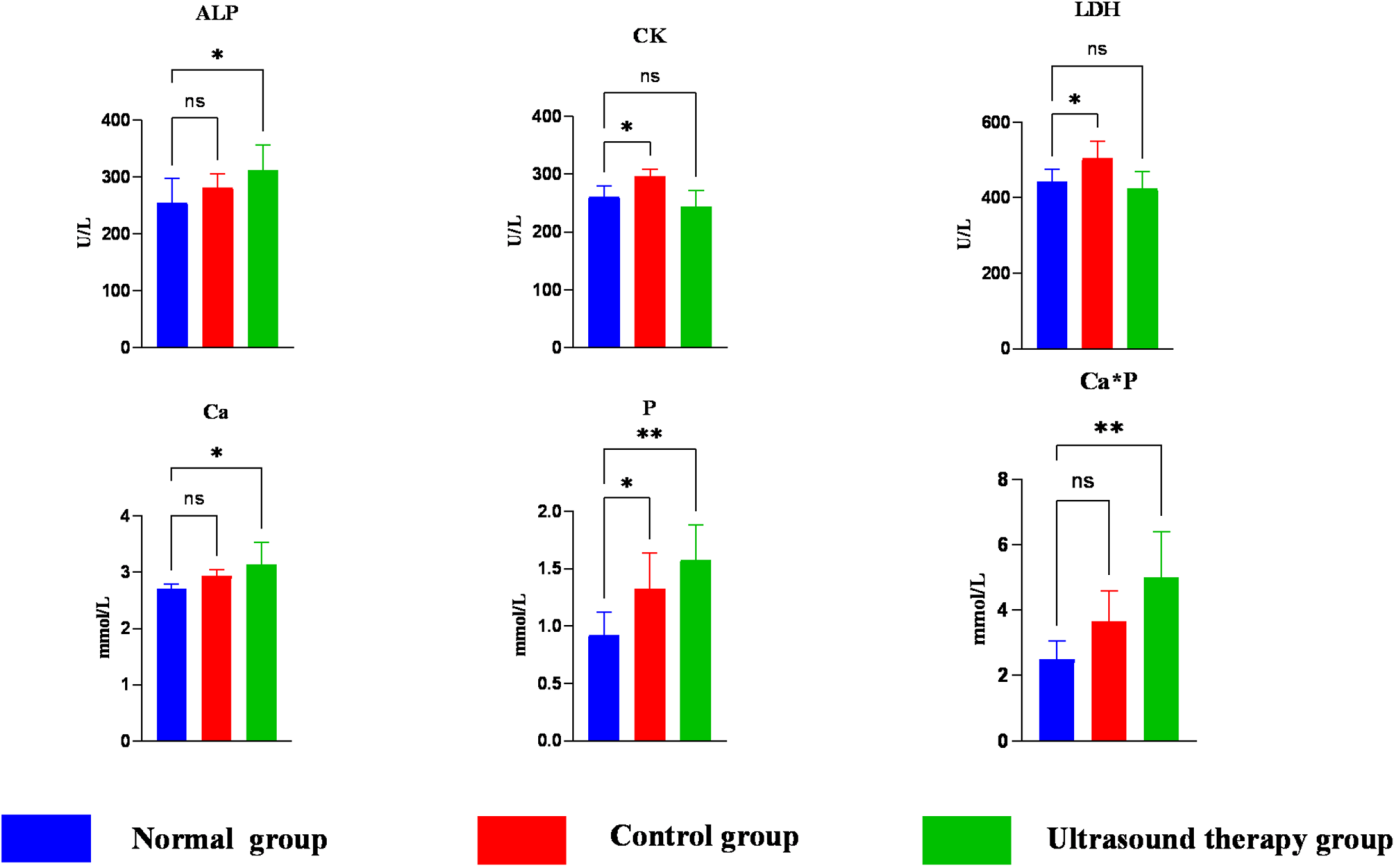
Differences in blood biochemical indices between groups. ALP, alkaline phosphatase; CK, creatine kinase; LDH, lactate dehydrogenase; Ca, calcium; P, inorganic phosphorus; Ca × P, calcium-phosphorus product. One-way ANOVA was performed to compare indices. In the bar graphs, asterisks indicate statistical significance: *P < 0.05; **P < 0.01; ***P < 0.001; ****P < 0.0001. In tables, lowercase superscripts denote *P* < 0.05 (significant difference); uppercase superscripts indicate *P* < 0.01 (highly significant difference); no superscript indicates *P* > 0.05 (no significant difference).

Following 4 weeks of LIPUS treatment, the ultrasound group exhibited significantly higher ALP and Ca levels (*P* < 0.05), and extremely significant increases in *P* and Ca × P (*P* < 0.01) compared to the normal group. CK and LDH levels showed no significant differences (*P* > 0.05) (Fig. 8).

### Inflammatory cytokine levels

Inflammatory cytokine results are presented in Table 5. Compared to the normal group, the control group showed significantly elevated levels of TNF-α, IL-1β, and IL-6 (*P* < 0.05).

**Table 5:** Results of inflammatory factor indexes in each group.

Projects	Normal group	Control group	Ultrasound therapy group
(TNF-α)/Pg/mL	237.9 ± 73.5	333.6 ± 24.8	246.6 ± 74.9
(IL-1β)/Pg/mL	359.2 ± 16.6	387.9 ± 18.6	379.1 ± 18.6
(IL-6)/Pg/mL	234.9 ± 22.8	307.8 ± 57.2	246.46 ± 27.4

**Fig. 9:**
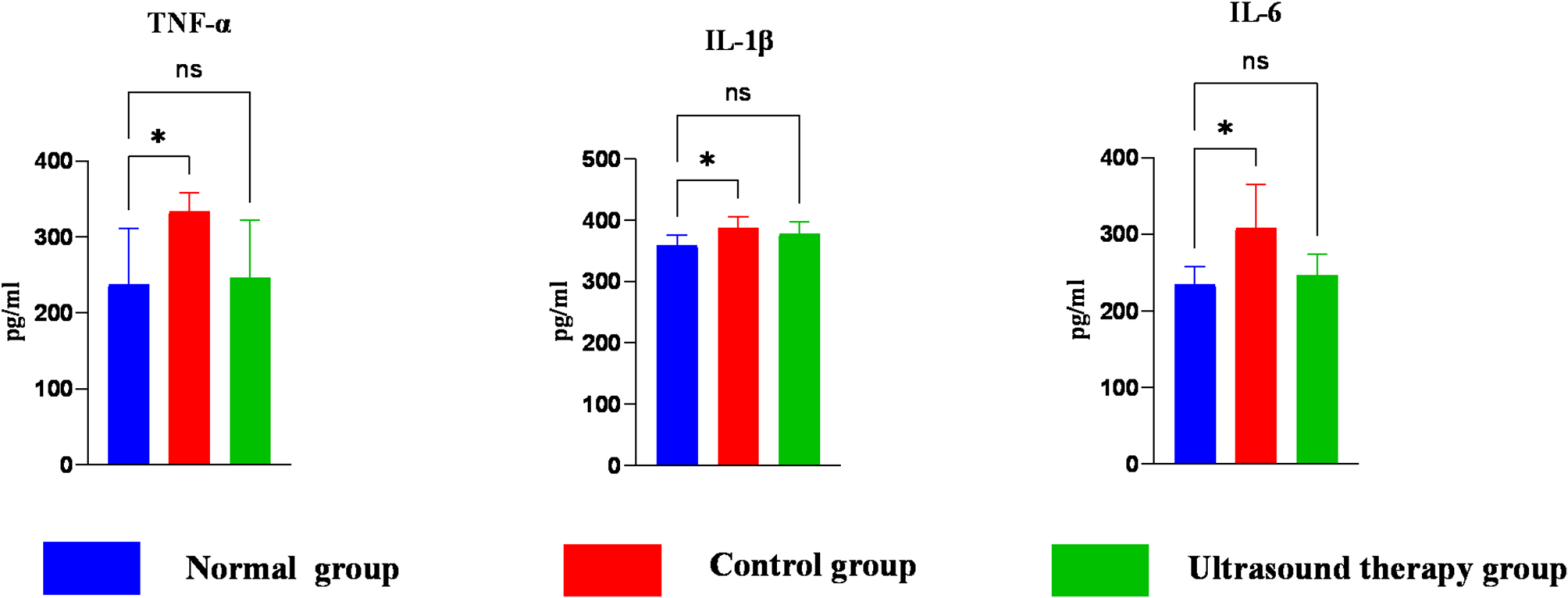
Differences in inflammatory factor indices between groups. TNF-α, tumor necrosis factor-α; IL-1β, interleukin-1β; IL-6:interleukin-6. One-way ANOVA was performed using to compare indices. In the bar graphs, asterisks indicate statistical significance: *P < 0.05; **P < 0.01; ***P < 0.001; ****P < 0.0001. In tables, lowercase superscripts denote *P* < 0.05 (significant difference); uppercase superscripts indicate *P* < 0.01 (highly significant difference); no superscript indicates *P* > 0.05 (no significant difference).

After 4 weeks of LIPUS treatment, no significant differences were observed in TNF-α, IL-1β, and IL-6 levels between the ultrasound group and the normal group (*P* > 0.05) (Fig. 9).

## Discussion

The sesamoid bones function with the flexor tendons during contraction at the palmar aspect of the fetlock joint and exercise-related PSBFs are among the most common catastrophic injuries in horses^21^. Normally. During intense exercise, excessive flexion of the fetlock joint causes the sesamoid bones to be compressed between the third metacarpal bone, the suspensory ligament, and the surrounding soft tissues. This increases pressure on the apical, lateral, and basilar regions of the sesamoid bones, compromising their blood supply, reducing bone density, and predisposing horses to PSBFs^22–24^. Studies have shown that racehorses with catastrophic proximal phalanx fractures have lower bone density and reduced subchondral bone thickness in the proximal phalanx compared to non-racing horses, with greater differences in bone density, possibly indicating a maladaptive response to exercise^25^. This partially explains why PSBFs occur more frequently in sport horses. In this study, all 13 Yili horses developed varying degrees of lameness during competitions held in the summer and autumn seasons and were ultimately diagnosed with PSBF through clinical and laboratory examinations. The data from these 13 cases provide a new perspective for lameness evaluation in Yili horses with PSBF, allowing for a more refined diagnostic approach. It was also observed that male Yili horses had a higher incidence of PSBF than females, and that forelimb involvement was more common than hindlimb.

PSBF can be preliminarily diagnosed through clinical history, inspection, palpation, and confirmed with diagnostic nerve blocks^26^. In this study, diagnosis was confirmed through a combination of clinical and laboratory assessments, with radiographic imaging playing a major role. The 13 diagnosed horses exhibited different types of sesamoid fractures, including apical, avulsion, mid-body, basilar, and mixed-type. Due to the anatomical specificity of equine proximal sesamoid bones, a comprehensive radiographic assessment typically requires three standard projections: lateral-medial (LM), dorsolateral-palmaromedial oblique (DL-PaMO, 45°), and palmarolateral-dorsomedial oblique (PaL-DMO, 45°). These imaging techniques provided essential diagnostic references for confirming PSBF in Yili horses.

Neutrophils and lymphocytes are two important hematological indicators, and studies have demonstrated that the neutrophil-to-lymphocyte ratio (NLR) is a useful systemic inflammatory marker for prognosis^27^. Hematocrit refers to the proportion of red blood cells in a given volume of anticoagulated whole blood after centrifugation, typically accounting for approximately 40% of the total blood volume under normal conditions. When the hemoglobin concentration falls below normal levels, it becomes difficult to maintain adequate blood volume and tissue oxygenation^28^. In the present study, hematological analysis of PSBF-affected Yili horses revealed elevated levels of neutrophils, white blood cells, and lymphocytes, suggesting that the fracture triggered a systemic inflammatory response. Decreases in red blood cell count, hemoglobin, mean platelet volume, and hematocrit indicate potential problems with impaired hematopoiesis, anemia, or dehydration.

Alkaline phosphatase (ALP) hydrolyzes organic phosphate compounds, releasing inorganic phosphate ions, which then combine with calcium ions to form calcium phosphate that is deposited in bone tissue. Therefore, changes in ALP levels can reflect the degree of fracture healing^29^. The interaction between serum calcium and phosphorus is critical. When the calcium-phosphorus product exceeds the solubility product of Ca₃(PO₄)₂ and CaCO₃, calcium phosphate first forms a colloid within the organic bone matrix and Ca₃(PO₄)₂ and CaCO₃, then precipitates as inorganic bone mineral content^30^. Thus, ALP, Ca, P, and Ca × P levels can serve as indicators of osteoblastic activity and fracture healing status. Creatine kinase (CK) and lactate dehydrogenase (LDH) are associated with organ function and muscle injury^31^. In this study, it was observed that treatment with LIPUS significantly increased the activity of ALP, Ca, P, and Ca × P. This suggests that the therapeutic effects of LIPUS may involve enhanced osteoblastic activity, as evidenced by increased ALP levels and elevated calcium-phosphorus product, thereby promoting calcium salt deposition and facilitating bone repair. Moreover, as PSBF is often associated with secondary tendon inflammation, the significant post-treatment reduction in serum CK and LDH levels could indicate effective mitigation of the inflammatory response and alleviation of muscle damage.

Inflammatory markers play an essential role in the diagnosis and treatment of various diseases. Studies have shown that the release of inflammatory cytokines such as TNF-α, IL-1β, and IL-6 during the acute inflammatory phase is critically important for early fracture healing^32,33^. During this phase, activated macrophages secrete a variety of cytokines, including IL-1β, which increases osteoclast activity and enhances osteoclast differentiation, thereby promoting bone resorption while inhibiting osteoblast synthesis^34^. TNF-α has a dual regulatory effect on bone homeostasis by promoting osteoclastogenesis on the one hand and stimulating osteoblast differentiation through the activation of the TNFR2 signaling pathway on the other^35^. In the early phase of fracture healing, IL-6 enhances neutrophil phagocytosis of cellular debris and stimulates osteoclast formation, which is beneficial for early healing. However, during the late healing phase, IL-6 promotes bone resorption, which may delay fracture healing^36^. The findings of the present study are consistent with previous research. Concentrations of TNF-α, IL-1β, and IL-6 in the serum of PSBF-affected horses were significantly elevated before treatment, indicating their active involvement in the early inflammatory response to PSBF. After treatment with LIPUS, the serum levels of TNF-α, IL-1β, and IL-6 returned to near control levels. Prior studies have confirmed that pro-inflammatory cytokines such as TNF-α, IL-1β, and IL-6 increase during the early phase of fracture and then gradually return to baseline levels, indicating resolution of inflammation and acceleration of the healing process^37^.

Current treatment strategies for PSBF include osteoclast inhibitors (e.g., tiludronate), intra-articular injections (e.g., triamcinolone into the metatarsophalangeal joint or platelet-rich plasma), orthopedic plates, and radial pressure wave therapy^38^. In cases involving small bone fragments, surgical removal is sometimes performed using minimally invasive arthroscopic surgery, but this approach can damage the ligaments surrounding the sesamoid bones^39^. Vascular supply is a critical factor in successful fracture healing, and Rawool et al.^40^ reported that LIPUS increased local blood flow at the fracture site thereby facilitating bone healing. In older horses, vascular sclerosis and slower metabolism result in reduced local blood circulation compared to younger horses. LIPUS supports repair by enhancing perfusion at the fracture site. Additionally, LIPUS promotes integrin expression, enhances osteoblast adhesion to the fracture site, and regulates gene expression, all of which contribute to improved healing^41,42^. As an evolving technology, LIPUS has been proven to accelerate bone healing while minimizing thermal effects. This non-invasive therapy facilitates osteogenesis through complex molecular, biological, and biomechanical interactions at the tissue and cellular levels^43^. In the present study, LIPUS was used to treat PSBF, effectively avoiding secondary infection and ligament damage that may result from surgical removal, while improving local blood supply and enhancing fracture healing in a safe and efficient manner.

## Conclusions

This study demonstrated that lameness scoring, radiographic imaging, and hematological analysis can accurately identify the cause and site of PSBF in horses, enabling precise diagnosis and targeted treatment based on the location of sesamoid bone injury. LIPUS therapy was found to enhance local blood supply at the fracture site, thereby accelerating bone healing and providing a safe and efficient treatment option for PSBF. Post-treatment evaluations using lameness scoring, hematological indices, and imaging further confirmed its therapeutic efficacy. These findings suggest that LIPUS has significant clinical potential for the treatment of PSBF and offers a safe, effective, and non-invasive alternative for equine fracture management.

## Materials and methods

### Study design

A randomized controlled animal trial.

### Time and location

The experiment was conducted from August 2023 to December 2024 at the Military Horse Farm in Zhaosu County, Ili Kazakh Autonomous Prefecture, Xinjiang Uygur Autonomous Region, China (Zhaosu, 43° 09′ N–43° 15′ N).

### Experimental materials

A fully automated five-part animal hematology analyzer (BC-5300VET, Mindray Animal Medical Technology Co., Ltd., Shenzhen, China), a fully automated biochemical analyzer (BS-240VET, Mindray Animal Medical Technology Co., Ltd., Shenzhen, China), an R103 portable DR system (Keda Instruments Co., Ltd., Dandong, China), lead aprons for radiation protection (Longyue Medical Company), a low-intensity pulsed ultrasound therapeutic device (HORSELIPUSPRO, Zhouquan Biotech Co., Ltd., Beijing, China), and equine-specific research ELISA kits for tumor necrosis factor-α (TNF-α), interleukin-1β (IL-1β), and interleukin-6 (IL-6), purchased from Yancheng Maiji Biomedical Testing Service Center.

### Experimental methods

Data collection included breed, age, sex, epidemiological history, affected area, clinical symptoms, imaging results, and hematological parameters, obtained through basic clinical diagnostic procedures to confirm the disease site and status for accurate diagnosis. Analysis and statistics of the collected information were used to formulate diagnostic strategies for PSBF in Yili horses. Diagnosis was based on clinical examination, standard lameness evaluation, local palpation, joint flexion tests, imaging, and hematological examinations to confirm the affected limb and fracture type.

Lameness was graded using a clinical lameness scoring system^44^ (Table S1), and lameness scores were compared before and after treatment to evaluate the horse’s quality of life and severity of injury.

All the animal experiments adhere to the ARRIVE guidelines (https://arriveguidelines.org) and were approved by the Animal Welfare and Ethics Committee of Xinjiang Agricultural University (Approval Number: 2024028; Date 27 December 2024). All methods were carried out in accordance with relevant guidelines and regulations.

### Experimental grouping

Twelve horses diagnosed with PSBF based on clinical and imaging evaluations were randomly divided into two groups: LIPUS treatment group and control group, with six horses in each group. Horses in the treatment group received LIPUS therapy, while those in the control group received no treatment. Additionally, six clinically healthy horses with similar physiological status and management conditions, and showing no signs of PSBF, were selected as the normal group for serum index analysis.

### LIPUS treatment protocol

Horses in the LIPUS treatment group underwent the following procedure: the affected limb was treated with ice packs or cold-water immersion once daily for 30 min, followed by elastic bandaging and fixation with gauze bandages. The horses were then rested for 45 days, with brief lead walking exercises on soft ground daily.

After 45 days of rest, LIPUS therapy was administered using an ultrasound probe with an effective radiating area (ERA) of 5 cm^2^, applied perpendicular to the affected fetlock. LIPUS parameters were set to a frequency of 3 MHz and a SATA intensity of 300–500 mW/cm^2^. Treatment was performed once daily for 25 min, at the same time each day, for a total of 28 sessions over 4 weeks (as illustrated in Fig. S1).

### Primary observation indices

#### Hematological parameters

A BC-5300Vet automated five-part animal hematology analyzer was used to evaluate hematological indicators including: white blood cells (WBC), neutrophils (Neu), lymphocytes (Lymph), monocytes (Mon), eosinophils (Eos), basophils (Bas), red blood cells (RBC), hemoglobin (HGB), hematocrit (HCT), and mean corpuscular volume (MPV), totaling 10 parameters.

#### Biochemical parameters

A BS-240VET automated biochemical analyzer was used to measure the following biochemical indicators: calcium (Ca), inorganic phosphorus (P), alkaline phosphatase (ALP), lactate dehydrogenase (LDH), creatine kinase (CK), and the calcium-phosphorus product (Ca × P), totaling 6 parameters.

#### Inflammatory markers

Equine-specific ELISA kits were used to assess serum inflammatory cytokines at the same time point in both treatment and control groups. The measured cytokines included tumor necrosis factor-α (TNF-α), interleukin-1β (IL-1β), and interleukin-6 (IL-6), totaling 3 parameters.

### Statistical analysis

Data were expressed as “mean ± standard deviation (mean ± SD).” One-way ANOVA was performed using GraphPad Prism™ software to compare indices before and after treatment. In the bar graphs, asterisks indicate statistical significance: *P < 0.05; **P < 0.01; ***P < 0.001; ****P < 0.0001. In tables, lowercase superscripts denote *P* < 0.05 (significant difference); uppercase superscripts indicate *P* < 0.01 (highly significant difference); no superscript indicates *P* > 0.05 (no significant difference).

## Supplementary Information

Supplementary Information 1.

Supplementary Information 2.

Supplementary Information 3.

Supplementary Information 4.

## Data Availability

Data is provided within the manuscript or supplementary information files. More details are available from the corresponding author upon reasonable request.
