# Supplementary material for: Clinical study on the effect of low-intensity pulsed ultrasound on healing of proximal sesamoid bone fractures in Yili horses
Source: Sci Rep. 2025 Aug 28;15:31697. doi: 10.1038/s41598-025-17424-0 (PMC12394538; doi:10.1038/s41598-025-17424-0)
Supplement: Supplementary file 3 — Supplementary Information 3. [file 41598_2025_17424_MOESM3_ESM.docx]

**Supplementary Materials**


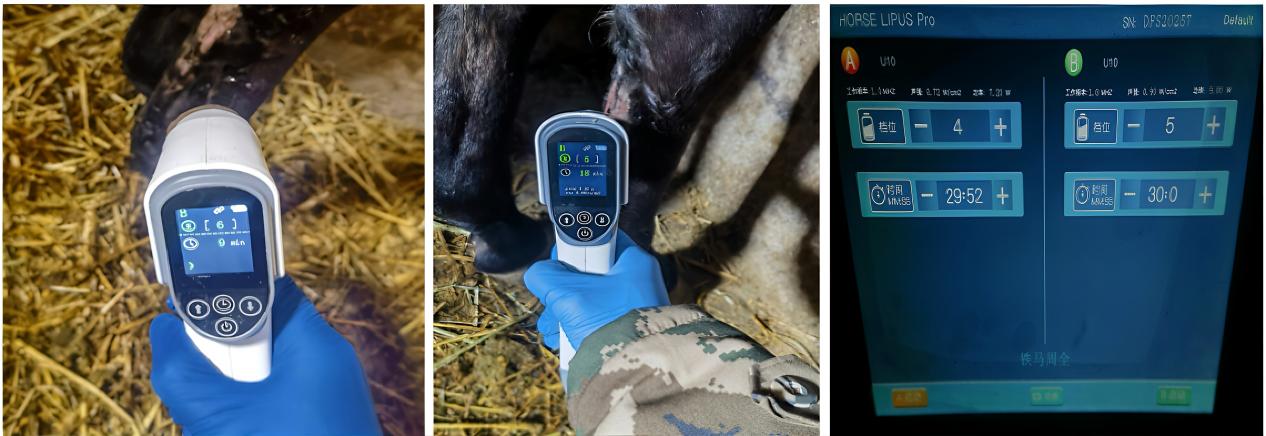


**Figure S1.** Low-intensity pulsed ultrasound therapy (LIPUS) for healing PSBF in Yili horses.

**Table S1.** Lameness grades and scores of horses after exercise.

| **Grade** | **Clinical manifestation** | **Score** |
| --- | --- | --- |
| **I.** Normal | No signs of lameness; normal gait. | 0 |
| **II.** Minor | Lameness is not obvious or is inconsistent; only observed under certain conditions such as under saddling or circling on hard ground. | 1 |
| **III.** Moderate | Lameness is not noticeable at a walk or straight-line trot but becomes obvious under specific conditions such as circling. | 2 |
| **IV.** Claudication | Lameness is clearly visible and persistent during a straight-line trot. | 3 |
| **V.** Severe | Lameness is apparent even at a walk. | 4 |
| **Ⅵ.** Grievous | The horse is unwilling or unable to bear weight on the affected limb or cannot walk. | 5 |
